# Planning during pregnancy

**DOI:** 10.7554/eLife.47985

**Published:** 2019-05-07

**Authors:** Bridget M Kuehn

**Affiliations:** eLifeChicagoUnited States

**Keywords:** scientist and parent, women in science, family friendly policies, diversity, careers in science, pregnancy

## Abstract

Colleagues, funders and institutions can support pregnant researchers in a variety of ways.

Navigating her own two pregnancies while running a laboratory taught Shubha Tole, now a senior professor at the Tata Institute of Fundamental Research in Mumbai, India, that pregnancy does not have to derail a woman’s career in science. Since then, she has helped three of her postdoctoral fellows navigate their own pregnancies while continuing their projects. “People come up with all kinds of creative solutions,” Tole says.

Institutions and funders often have policies to support pregnant researchers, including paid family leave, laboratory safety precautions, and funding for technicians to assist with research tasks. But help can also come from colleagues being flexible to accommodate medical appointments or childcare pick-ups, or simply lending a helping hand when unforeseen hiccups arise.

Jillian Nissen had her first child when she was a postdoctoral fellow at Stony Brook University in New York, United States, where she used mice to study multiple sclerosis. She found the smell in the animal facility nauseating during her first trimester. “Having a really good supportive [project advisor] and group of peers helped with those challenges,” she says. Nissen's colleagues helped her with small tasks in the animal facility and with any lifting she had to do.

The first trimester of pregnancy – before many women feel comfortable disclosing their pregnancy – can be the most vulnerable time

## Safety first

Making sure the research environment is safe for mother and baby is crucial throughout pregnancy. The National Postdoctoral Association’s Guide to Pregnancy and Maternity Leave lists some types of research and research tools that may pose risks to a developing fetus, including anesthesia, radiation, chemicals and solvents, exposure to loud noises or vibrations, and strenuous physical activities involved in field work or laboratory work. The guide recommends that women discuss their work and its potential risks with their doctor.

“There were some chemicals that we were using in the labs that I had to be a lot more cautious around,” explains Nissen, who consulted her physician and did research on her own. “I got a lot more careful about masks and gloves with certain things that I normally wouldn’t have worried about.”

Kathleen Flint Ehm, who co-authored the NPA pregnancy guide and is now director for graduate and postdoctoral professional development at Stony Brook University, recommends that women who are trying to become pregnant contact the environmental health and safety office at their institution for a safety review. The first trimester of pregnancy – before many women feel comfortable disclosing their pregnancy – can be the most vulnerable time so it helps to know the risks beforehand.

A separate Pregnancy Lab has been established at the Research Institute of Molecular Pathology (IMP) in Vienna, Austria, to give pregnant and nursing mothers a place where they can continue research without potentially being exposed to harmful substances. About 10 women make use of the Pregnancy Lab each year, says Harold Isemann, managing director of finance and administration at IMP. The lab contains all the necessary scientific equipment and a member of the institute’s biological safety staff makes sure that protocols stay within chemical exposure limits set by the Austrian government. Technicians complete any tasks that exceed the limits. “[Researchers] can basically continue with their work as soon as they report their pregnancy,” Isemann explains.

Carolin Charlotte Wendling, then a postdoctoral fellow at the Geomar marine biology institute in Kiel, Germany, also relied on a technician during her pregnancy. Wendling, who works with marine microbes, found that strict regulations in Germany prohibited her from working with pathogens during pregnancy. A staff safety officer determined what percentage of her work she would be able to complete under these rules and the institution’s health insurance covered the costs of hiring a technician to complete the rest.

“I could still work in front of the computer, analyze data, write up any manuscripts,” Wendling explains. “It did not delay my research too much. Overall, I think I was really lucky.”

**Figure 1:**
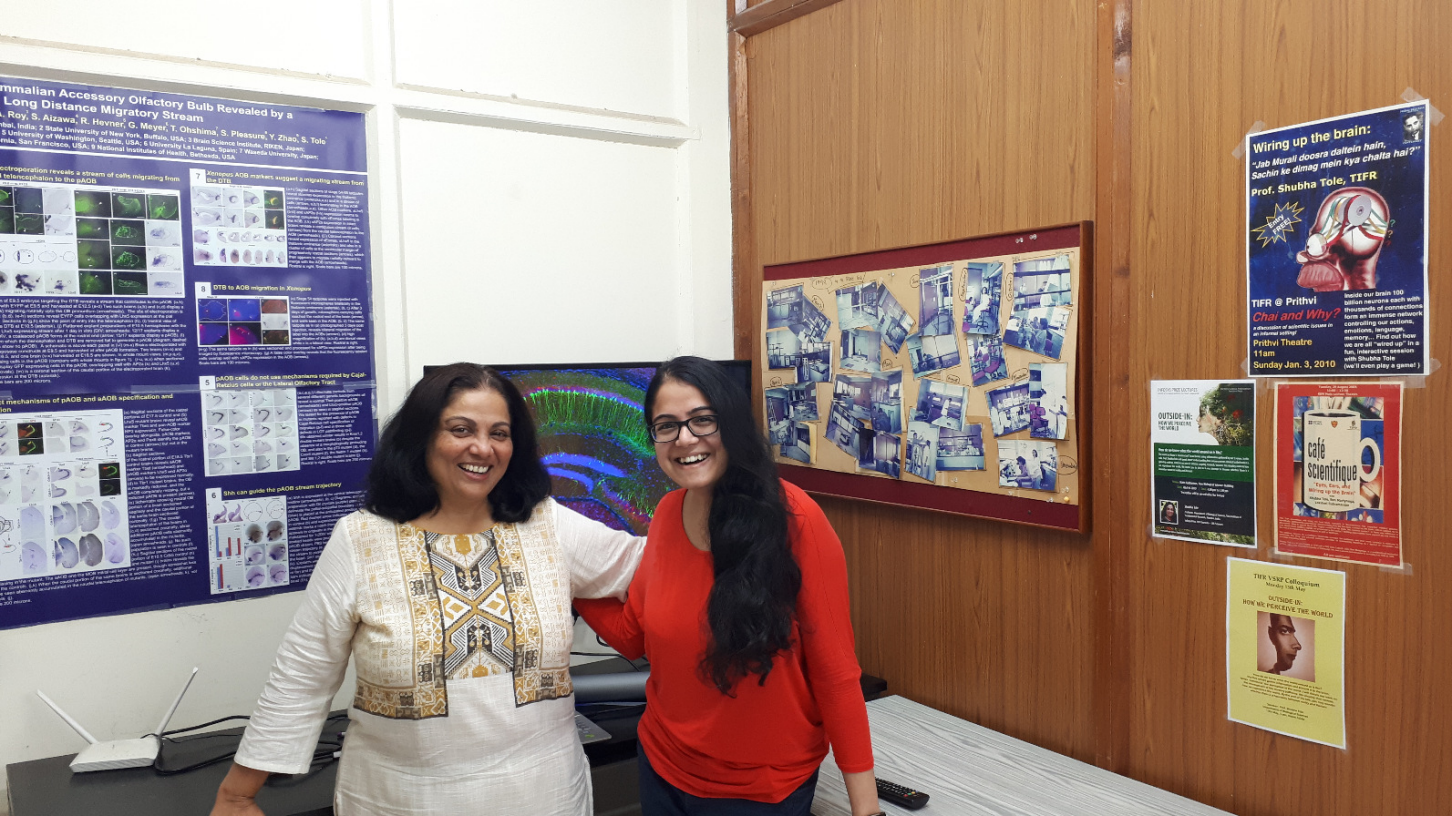
Shubha Tole (left) with her postdoctoral fellow Archana Iyer in the lab. Iyer has just returned after six months of maternity leave. During this period of leave, Bhavna Pydah was hired as a technician to provide research support.

Photograph: Bhavna Pydah.

## From supported to supportive

Tole and her husband chose to delay having their children until she felt established in her laboratory: “I couldn't grow two things at once!”

When she had her first child, her group members came to lab meetings at her on-campus home during her 4.5 month paid maternity leave (India has since adopted a six month paid leave). India’s maternity leave policy allows women to work from home if they wish to, and Tole took advantage of this: “I wrote two papers during my maternity leave, so that was extremely productive.”

During Tole’s pregnancy, senior staff expressed support for her and her husband (a scientist who also took parental leave), and they allowed her to forego an annual staff trip. Now, she tries to make sure her lab members feel as supported as she did. She is flexible about when they are in the lab, how long their leave is (and whether they want to work during it), or when they need to take time away for childcare. This can lead to unconventional solutions: one postdoc, whose husband was working in England, hired a night nanny and opted to burn the midnight oil imaging at a microscope to allow her to care for her baby during the day.

“When a postdoc tells me that she is pregnant, I hire a technician to support her for the coming two years,” Tole says. “The goal is to enable a talented scientist to continue her science as best as she wishes. There’s no point in stressing out a new mom.”

## Planning and policies

One of the things many women and leaders in the field agree on is that planning is essential for successfully managing research and pregnancy. Ehm recommends that postdoctoral researchers create a research plan during their pregnancy and discuss it with their colleagues. “It engages your collaborators and your advisor in a productive way that shows them that while there may be concerns about productivity, you do have a plan,” she explains.

Plans should take into account the legal entitlements that many countries provide to pregnant employees. In the United States, Title IX affords women protections during their pregnancy and maternity leave (for more detail, see The Pregnant Scholar), and most employees are guaranteed 12 weeks of unpaid maternal leave. However, institutions may or may not offer paid parental leave, notes Ehm. When Nissen was a postdoctoral fellow, she did not have access to paid maternal leave – a perk that has only been added at Stony Brook in the last year for postdoctoral fellows who are employees. Instead, she used vacation and sick days. Some universities may also allow women to work part-time to help stretch their maternity leave.

Nissen found it essential to plan carefully for the periods before and after her leave. She made sure her animal experiments would be wrapped up two weeks before her due date, and asked colleagues to set aside animals for her during her maternity leave so she could pick up her work when she returned. She also advises planning where to pump breast milk on returning to work. Some institutions like IMP offer onsite childcare, which allows mothers to nurse their child during the day instead of pumping; others may have designated spaces for pumping and storing breast milk. Nissen had to use her advisor’s office when she returned. She also had to plan her experiments around the times when she would need to pump, and make sure things were wrapped up in time for her to pick up her child from daycare.

During her second pregnancy, Nissen ran a lab of her own at the State University of New York at Old Westbury. She had to temporarily shut down the lab when she was on maternity leave because the undergraduate students working in her lab could not continue without supervision. “Pregnancy and childbirth definitely derailed my lab work much more during my time as a faculty member,” she says. “As a postdoc, I had to put my own individual project on hold once I gave birth, but thanks to my group of peers I could seamlessly restart my work as soon as I returned. As a lab leader, I had to shut down all of the experiments a few weeks prior to my due date and I was not able to get things running again until after I had returned to work.”

Some funding organizations have policies to help new and expecting mothers. For example, the Welcome Trust/DBT India Alliance has stopped counting the time that grantees spend away from research in their fellowships and they provide a one-year extension in funds for women who take maternity leave. So far about 11 women have used the maternity extension. “The feedback is extremely positive, with women fellows telling us how transformative this has been for their research careers,” says Shahid Jameel, the organization’s chief executive officer. “More than the funds, it is the comfort of having the organization back them up with a policy.” Other funders, including the National Science Foundation in the United States, cover the costs of hiring a technician to continue a fellow’s work during their leave.

Although they didn't always say it outright, most male scientists were not eager to take on women because they didn't think they would be 100% focused on projects

## Systems of support

Nearly half of women and about one-quarter of men in the US leave full-time employment in science after their first child is born, according to a recent study. But there are many ways governments or employers can reduce this attrition.

During her tenure as the first woman dean of the graduate division at the University of California, Berkeley between 2000 and 2007, Mary Ann Mason found that many women were leaving during their postdoctoral years. And some faculty were not supportive of pregnant researchers. “Although they didn't always say it outright, most male scientists were not eager to take on women because they didn't think they would be 100% focused on projects, and science is very competitive.”

Mason argued that protections for pregnancy were required under Title IX and established a policy that gave both men and women four months off for parental leave, during which time the tenure clock is stopped. “That was very, very popular,” Mason says. “We had a lot of people get tenure who wouldn't have gotten tenure.”

The policies eventually spread to other University of California (UC) campuses. Mason noted that in the five years following the passage of these policies the rate of childbirth doubled among faculty and postdoctoral students in the UC system. Now, UC is looking to tackle another big challenge for scientist parents: childcare. This includes offering on-campus and emergency childcare, and childcare stipends.

The state of California in 2014 passed a law protecting students from pregnancy discrimination and guaranteeing graduate students the opportunity to take a leave of absence for childbirth and return in good academic standing. Ehm believes that having standardized state or national policies can help reduce the burden on both the pregnant scientist and their advisor: “It should just be a guaranteed thing that you get, and you shouldn't have [to negotiate] on a case-by-case basis.”

Many accommodations for researchers who are pregnant or new parents cost employers and advisors nothing. For example, Deepak Modi, head of the Molecular and Cellular Biology Laboratory at the National Institute for Research in Reproductive Health in Mumbai, India, has allowed lab members to shift their hours or work from home to accommodate child care needs. He’s also worked with conference organizers to allow a student to bring her nanny to a meeting for no additional cost.

Modi acknowledges that he was not aware of the challenges pregnant researchers face the first time a student approached him about her pregnancy, but he was still able to make accommodations. The second time around, the process went more smoothly because he hired a technician to continue the student’s experiments during leave, allowing her work to continue to progress.

Now, he advocates for his colleagues to adopt more supportive attitudes toward researchers who get married or have children. “These should not be considered as hindrances to the research program,” Modi says.

## A question of timing

Though many women wait until after 12 weeks to notify their advisor or colleagues about their pregnancy, Nissen suggests that letting them know earlier can be helpful. “If you have a good relationship with your advisor, then don't be afraid to tell them earlier rather than late,” she says. “It'll help them understand what's going on for you and help them accommodate your doctor's appointments or any medical issues in that first trimester, which a lot of people have.”

Wendling advises women not get too hung up on the timing of their pregnancies during their career: there are many ways to manage pregnancy while continuing your research. “Never wait for the right time,” she says. “The right time is not coming.”

## Note

This Feature Article is part of the Scientist and Parent collection.

